# Comparison Between Photofluorography and Standard Fluoroscopic Voiding Cystourethrography in Evaluating Vesicoureteral Reflux in Children With Urinary Tract Infection

**DOI:** 10.5812/numonthly.3562

**Published:** 2012-06-20

**Authors:** Seid Ali Alamdaran, Mitra Naseri, Ali Beheshtian

**Affiliations:** 1 Radiology Department, Dr Sheikh Children Hospital, Mashhad University of Medical Sciences, Mashhad, IR Iran; 2 Pediatric Nephrology Departments, Dr Sheikh Children Hospital, Mashhad University of Medical Sciences, Mashhad, IR Iran; 3 Mashhad University of Medical Sciences, Mashhad, IR Iran

**Keywords:** Photofluorography, Vesico-Ureteral Reflux, Radiation Dosage

## Abstract

**Background:**

Imaging of the urinary system is considered to be responsible for significant radiation in children.

**Objectives:**

This study was conducted to measure and compare the radiation dose in spot films with photofluorography voiding cystourethrography (VCUG) in children.

**Patients and Methods:**

111 [222 Kidney Urinary Unit (KUU)] pediatric patients, aged 1 month to 5 years, with symptomatic urinary tract infection were enrolled in the study. Peak tube voltage (kVp), exposure setting (mAs), focus film distance (FFD), film size and DAP (after the exam) were recorded for all patients. To evaluate the validity of the photographs, we calculated sensitivity, specificity, predictive values and agreement between the two methods using the kappa statistic. If the kappa was greater than 0.75, between 0.4–0.75 or less than 0.4, then the agreement was excellent, good or poor, respectively. P values less than 0.05 were statistically significant.

**Results:**

Vesicoureteral reflux (VUR) was detected in 74 KUU (33.3%) in standard films and in 71 (32%) in photographic images. The photographs had no false positives and 3 false negatives. Therefore, the new method had a sensitivity of 96%, a specificity of 100%, a negative predictive value of 98% and a positive predictive value of 100%. The two-method agreement in the VUR diagnosis for grades 1, 4, 5 and the overall grading were excellent (kappa > 0.83); however, for grades 2 and 3, agreement was 80%, which was good (kappa = 0.64).

**Conclusions:**

Our study suggests that the high validity and excellent agreement of the photofluorography method in the diagnosis and grading of VUR, which is comparable to spot films and represents a 50%–90% reduction in radiation, makes it the preferred method.

## 1. Background

In the 11th Report on Carcinogens of the United States National Toxicology program, X-irradiation and ionizing irradiation were placed on the list of known human carcinogens (1). Imaging of the urinary system is considered responsible for about one-quarter of the genetically significant radiation exposure in children. At present, the gold standard for diagnosis and grade determination of vesicoureteral reflux (VUR) is fluoroscopic voiding cystourethrography (VCUG) with spot films because of its acceptable sensitivity and negative predictive value (2-4).

In addition to demonstrating anatomic abnormalities of the urinary tract, the examination provides a physiological means of detecting and characterizing VUR. It is estimated that the incidence of urinary tract infection (UTI) during childhood is 8% in girls and 2% in boys (5). The amount of ionizing radiation delivered to a child during a VCUG examination is less than that delivered during other commonly performed pediatric examinations that involve ionizing radiation, such as computed tomography. Nevertheless, diagnostic information combined with minimal radiation exposure is a constant concern when employing radiographic techniques, especially in children. According to ALARA concept, radiation exposure during VCUG should be kept to a minimum. The ALARA concept is especially important in children because their rapidly developing tissues and organs are approximately 10 times more sensitive to ionizing radiation than middle-aged adults (6).

Conventional fluoroscopy employs a relatively large radiation dose, and approximately 25% of the genetically significant radiation dose in children arises from imaging of the urinary tract. Generally, the fluoroscopic dose is estimated to be 80% of the entire radiation dose in a VCUG examination.

We guess that photofluorography with hard copy images without using spot film would result in a diagnostic quality comparable to that of standard fluoroscopic VCUG for detection of VUR in childhood UTI. The benefit of this method may be lower radiation doses.

## 2. Objectives

This study was conducted to measure and compare radiation doses in spot films with photofluorography VCUG in children.

## 3. Patients and Methods

Data were collected from the pediatric radiology department of an academic center (Dr. Sheikh Children’s Hospital, Mashhad University of Medical Sciences). The enrolled population included 111 (222 KUU) pediatric patients, aged 1 month to 5 years (mean 2 years and 4 months), with histories of documented symptomaticUTI. Urinary tract infection was defined as growth of more than 100,000 colony-forming units/mL of one microorganism in cultured urine. Urine samples were obtained by mid-stream collection (toilet-trained patients) or urine bags (non-toilet trained patients). Radiographic parameters, such as peak tube voltage (kVp), exposure setting (mAs), focus film distance (FFD), film size and DAP (after the exam), were recorded. Based on NRPB recommendations, there were only minor differences between the DAPs of the 1and 5-year-old groups (7). Therefore, patients under 5 years of age with UTI who were referred for VCUG were studied. All studies were performed on an Appelem Radiography/fluoroscopy unit.

DAPs were recorded in units of mGycm2 using an 841-c meter (Gammex), which is specifically sensitive enough for pediatric studies. Our fluoroscopy unit has a 3.2 mm aluminum equivalent total filtration, and the FFD was 110 cm for all patients, which was operated at 63–75 kVp, depending on the size of the patient. An image intensifier television chain was used for fluoroscopy, and a Sony CRs 105 mm camera was used to document most of the imaging. It should be noted that computed radiography (CR) (Agfa and Radlink Sony printers) was used for all examinations. Due to the fact that this equipment was newly installed in the department, so for both CR, the radiographers used the same settings for the VCUG examination.

The dose area product (DAP) is measured with an ionization chamber mounted directly to the light beam diaphragm housing. The DAP is defined as the absorbed radiation dose to air (or the air KERMA) averaged over the area of the X-ray beam in a plane perpendicular to the beam axis, multiplied by the area of the beam in the same plane. It is usually expressed in Gycm^2^ and is conveniently measured with special large-area ionization chambers (DAP meters) attached to the diaphragm housing of the X-ray tube, which intercepts the entire cross section of the beam. The meter device measures the total diagnostic DAP during radiography and fluoroscopy. This meter provides real time DAP measurements as well as total dose measurements. The values obtained with the DAP meter correspond to the absorbed skin dose over a specified surface area, reported as DAP. Measurements were done with both systems for conventional spot film and photofluorography.

Thermoluminescent dosimeters (TLDs) were prepared in plastic sachets and then used for monitoring exposure to the hands of parents who were asked to hold their child steady. We also used TLDs for background radiation measurements. Each sachet was labeled for left and right hands. ESD was measured directly by LiF:Mg,Ti thermoluminescent dosimeters (type TLD-100). Two TLDs were placed inside plastic sachets and attached to the skin on the back of the parents’ hands. The mean value for the two calculated ESDs was used as the measured dose in the hands. The TLD-100 LiF chips were annealed by heating at 400°C for 1 h, cooled slowly to ambient temperature and then reheated to 75°C and kept at that temperature for 18 h. These chips were then read using a Harshaw 3500 TLD Reader.

The amount of contrast media solution (30%) to be infused into the bladder was determined by predicting bladder capacity, which was estimated in milliliters using the following formulas: for children younger than 1 year, capacity = weight (kg) × 7; and for those older than 1 year, capacity = (age (y) + 2 )× 30). We modified the VCUG protocol (two photofluorography spot films of the urinary system during voiding in two left posterior and right posterior oblique positions). The parents helped to tabilize and support the children and catheter. We trained the parents to help and prevent undesired voiding by pushing or closing the children ’s external genitalia by their hands. Each study consisted of 3 to 4 digital radiographic films and synchronous fluoroscopic images printed on glossy paper. We had two types of images for comparison, radiographic spot images on film and identical paper images.

Photographic and spot radiographic images were then interpreted by two independent radiologists with at least 3 years of experience. For dosimetry, we chose 30 patients to compare radiation doses in both methods. These reports were collected and statistically analyzed using the appropriate tests from the SPSS 13 software package. To evaluate the validity of the photography, we calculated sensitivity, specificity, predictive values and agreement of two methods using the kappa statistic. Kappa greater than 0.75, between 0.4–0.75 or less than 0.4 were considered as excellent, good and poor, respectively. P values < 0.05 were reported as statistically significant.

## 4. Results

We analyzed 222 KUU (kidney urinary unit) reports for presence or absence of VUR and its grading in spot films (the gold standard) and photofluorography images. As a result, reflux was seen in 74 KUU (33.3%) in standard films and 71 (32%) in photographic images. The photographs had no false positives, wherase it had 3 false negatives results. According to our results the new method had a sensitivity, specificity, negative and positive predictive values of 96%, 100%, 98% and 100% respectively. The two-method agreement in the VUR diagnosis in grades 1, 4 and 5 and the overall grading were excellent (kappa > 0.83). However, in grades 2 and 3, the agreement was 80%, which was defined as good (kappa = 0.64).

In one case, the photography graded it higher (2→3) than standard spot films, and in seven patients, the photography graded it lower (3→2). In addition, the agreement in the diagnosis of VUR between the 2 radiologists 'reports was 100%, and the agreement in grading was 89% (kappa = 0.77 :excellen). Table 1 compares the results of the DAP values between the two methods. AS Table 2 shows DAP in photofluorography was lower than in spot film and the radiation dose increased based on body size. The parent’s hands were also within 2–25 cm of the main beam. According to our results, the radiation doses received by the parents were at background radiation levels.

**Table 1:** DAP^a^ of the VCUG a Data for the Two Methods (Spot Film and Photofluorography)

**Patient, No.**	**Age Group, y**	**Weight, kg, mean**	**Spot Film DAP, mGycm^2^**	**Photofluorography DAP, mGycm^2^**
9	0 < 1	6.4	56.25	10.75
21	1–5	12.2	97.13	11.38

^a^Abbreviations: DAP, dose area product; VCUG, voiding cystourethrography

**Table 2:** Comparison of DAP^a^ Values from This Study and VCUG a Standard in Other Studies (DAP Unit is Gycm^2^)

**Age Groups, y**	**Weight, kg**	**Almen and Mattsson ** **(14)**	**Martin ** **et al. (15)**	**UK**	**Persliden ** **et al. (13)**	**Spot Film (This Study)**	**Photofluorography (This Study)**
0 < 1	< 10	-	0.17	0.3	0.04–2.48	0.05	0.01
1–5	10 < 20	1.4	0.15	0.8	0.10–1.47	0.09	0.01

^a^Abbreviations: DAP, dose area product; VCUG, voiding cystourethrography

## 5. Discussion

There is a strong association between UTI and VUR in children and VUR is a main risk factor for renal scarring and renal failure in children. Hence, early diagnosis of urological anomalies mainly VUR is necessary to prevent late omplications of UTI in children. In assessing and grading vesicoureteral reflux, fluoroscopic VCUG with spot film is the current gold standard method; however, high radiation doses, especially with non-digital and non-pulsed fluoroscopes, are a problem. Studies have shown that elimination of spot film (i.e., digital or computer-based video frame fluoroscopy, the capture and delivery of fluoroscopic images to monitors or hard copies) reduces the radiation dose by 40%–60% (8-12).

In the course of time with development equipment of radiation systems, all searches directed to reduce radiation dose, especially in children (most sensitive and more life expanse for effect of radiation), without reducing the sensivity of method. very articles have shown that 50% or more portion of radiation belong to spot films (5, 8, 11, 12). Photofluorography is a new modified method that uses printed-paper images from a fluoroscope without the need for spot films.

In our study, the agreement and grade of the VUR diagnosis were excellent, and the sensitivity and negative predictive values were high. In addition, the reliability of the photographic images was high. The reliability was probably due to the significant agreement between the 2 radiologist reports in this study and the reports from other studies. The study probability could be repeated with the same results, and thus repeatability was high (10). The results of this study show that VCUG with photofluorography hard copy has a high diagnostic value with very low radiation doses (50%–90% reduction in radiation dose). In order to achieve quality paper images (photofluorography), we must be careful to regulate the contrast, brightness and sharpness of the fluoroscopic monitor and printer. However, we must accept the fact that the quality and resolution of paper images and photographs are lower than those of images on radiographic spot films, but they cannot influence the VUR diagnosis or determine its grade.

The comparison of DAP values obtained in this study with those taken from the literature is shown in Table 2. As this table demonstrates, our values are lower than those achieved by other studies. This is due to three issues: the equipment, the examination method and the non-use of fluoroscopy. DAP values from spot films are comparable with the Persliden results (13); this may be related to the use of computed radiography in our study and digital radiology in the Persliden study. On the other hand, our results were lower than the Almen and Mattsson studies and lower than the DRL (Diagnostic Reference Levels) in the UK. This is due to the use of the film-screen combination by the Almen, Mattsson (14) and UK studies. When comparing the values of this study with those of other studies, it is evident that the digital unit delivers lower radiation doses.

However, the authors did state that there was no significant difference in the interpretation of the images between the two types of images. The result of the present study supports this statement. Therefore, this study suggests that standard VCUG for reflux diagnosis and grading can be replaced with the photographic method. The outcome of this study shows that the examination technique in pediatric radiology is not yet optimized and that the non-optimized procedures contribute to considerable variations in radiation doses for children. According to our results, the radiation doses received by the parents were similar to background levels. The results could have been improved by using more sensitive TLDs, such as calcium sulfate TLDs, for measuring radiation to the parent’s hands because they are approximately 30 times more sensitive than (LiF) TLDs.

Although the doses received in fluoroscopy are lower than the dose equivalent limit recommended for the general public, exposure should be kept to a minimum, following the ALARA principle. However, the risk versus benefit of each radiograph is important and must be considered carefully, especially since radiation effects are cumulative. The results of this study show that standard VCUG for reflux diagnosis and grading can be replaced with the photographic method without spot films, although further studies using the same design are warranted. Quantitative methods for the assessment of patient doses should be implemented in radiology departments. More laboratory and clinical research is necessary to investigate methods for reducing radiation exposure during VCUG.

Our study suggests that the high validity and excellent agreement of the photofluorography method in the diagnosis and grading of VUR is comparable to that of spot films and also provides a 50%–90% reduction in radiation, making it the preferred method.
