# Corrigendum to: Comparison Between Photofluorography and Standard Fluoroscopic Voiding Cystourethrography in Evaluating Vesicoureteral Reflux in Children With Urinary Tract Infection [Nephro Urol Mon.2012;4(3): 541-544. DOI: 10.5812/numonthly.3562]

**DOI:** 10.5812/numonthly.13590

**Published:** 2013-09-15

The title of the manuscript should be changed to “A Comparison Between Patient Dose Arising From Photofluorographic and Standard Fluoroscopic Voiding Cystourethro Graphy in Children With Urinary Tract Infection“.

Malakeh Malekzadeh and Mohammad Taghi Bahreyni Toossi were incorrectly omitted as author and co-author respectively. Mohammad Taghi Bahreyni Toossi is the new corresponding author of the article. The authors and affiliations are listed as below:

Malakeh Malekzadeh ^1^, Mohammad Taghi Bahreyni Toossi ^2,*^, Seyed Ali Alamdaran ^3, ^Mitra Naseri ^4^, Ali Beheshtian ^5^

^1^ Nursing and Allied Health Faculty, Semnan University of Medical Sciences, Semnan, IR Iran

^2^ Medical Physics Research Center, Medical Physics Department, Faculty of Medicine, Mashhad University of Medical Sciences, Mashhad, IR Iran

^3 ^Radiology Department, Dr Sheikh Children Hospital, Mashhad University of Medical Sciences, Mashhad, IR Iran

^4 ^Pediatric Nephrology Department, Dr Sheikh Children Hospital, Mashhad University of Medical Sciences, Mashhad, IR Iran

^5^ Mashhad University of Medical Sciences, Mashhad, IR Iran

* Corresponding author: Mohammad Taghi Bahreyni Toossi, Medical Physics Research Center, Medical Physics Department, Faculty of Medicine, Mashhad University of Medical Sciences, Mashhad, IR Iran. Tel:0511-8828576, Fax: 0511-8002320, E-mail: bahreynimt@mums.ac.ir

The following sentence should be added to the end of the result in the abstract:

“The results of this study are evident that VCUG with photofluorographic hard-copy provides high diagnostic value with very low radiation dose to patients (patient dose reduced by 5-9 folds).”

In the abstract in line 3 of conclusions “50-90%” should be changed to “80-89%”:

“our study suggests that the high validity and excellent agreement of the photofluorography method in the diagnosis and grading of VUR, which is comparable to spot films and represents a **80-89%** reduction in radiation, makes it the preferred method.”

In the reference part reference 1 should be replaced with the following one:

Schumacher R and Allmendinger H. Optimization of pulsed fluoroscopy in pediatric radiology using voiding cystourethrography as an example. *MedicaMundi* 2008;52:18-24.

Inaddition, references 13, 14 and 15 are related to Persliden et al. , Almen et al. and Martin et al. respectively.

Finally, the author would like to acknowledge Miss Golsa Tabatabaei for editing this manuscript and all staff members in the studied centers which without their assistance this work may not have been accomplished. We also extend our thanks to the office of vice president for research of Mashhad University of Medical Sciences for funding this work.

